# Environmental regulation and green innovation of polluting firms in China

**DOI:** 10.1371/journal.pone.0281303

**Published:** 2023-03-09

**Authors:** Lingyan He, Miao Wang

**Affiliations:** 1 School of Banking and Finance, University of International Business and Economics, Beijing, China; 2 Centre for English Language Education, UNNC-NFTZ Blockchain Laboratory, University of Nottingham Ningbo China, Ningbo, Zhejiang, China; Yunnan Technology and Business University, CHINA

## Abstract

The main objective of this paper is to study the impact of the *Ambient Air Quality Standard (2012)* on the green innovation of Chinese firms in polluting industries. The analysis features “leverage effect” of Porter Hypothesis imposed by environmental regulations and exploits exogenous variations caused by the promulgation of the new policy. Based on the exogenous variations, this paper uses the time varying PSM-DID method. The findings of this study suggest that the implementation of the new policy improves firms’ green innovation. Increments in R&D investment and environmental protection investment are channels through which the new standard positively affects firms’ green innovation. The cross-sectional heterogeneity analysis exhibits that the effect of this environmental regulation is stronger for firms with bigger size and lower financial constraints. The contribution and significance of this study are as follows: our study enriches understanding of the impact of environmental regulation on firms’ green innovation by empirically confirming the influencing channels of the impact of environmental regulations on green innovation. In addition, this paper contributes to the firms’ green innovation literature by empirically validating the role of corporate characteristics in moderating the effect of environmental regulations.

## Introduction

China has become the world’s second largest economy owning to the rapid economic growth and economic transformation in the last four decades. Together with the high-speed development, China also has faced with the sever problem of pollutant emissions, among them, one of the most serious pollution problems would be carbon emission [1]. Air pollution has already become the main threaten of citizen’s health. According to Kahn and Yardley (2007) [2], only 1% of urban dwellers had clean air to breath according to European Union standard. In 2010, 1.2 million premature deaths in China were related to ambient PM2.5, which accounting for nearly 35% of such deaths worldwide [3].

Actually, the *2020 Global Environmental Performance Index (EPI) Report* shows that among the 180 countries and regions participating in the evaluation, China ranks 120^th^ which highlights the seriousness of China’s current environmental governance and relatively weak environmental protection. China’s central government faces with serious challenges both economically and politically. On the one hand, although the manufacturing industry has the high level of pollution, millions of Chinese citizens rely on it to “shaken off poverty” [4]. Specially, the rapid economic and industrial growth with comparatively low energy efficiency labeled China as the world’s largest energy consumer and carbon emitter [5]. To meet the growing energy demands, China highly depends on coal consumption [6]. Manufacturing industries, such as pressing and smelting of metals, manufacturing of non-metal mineral products and manufacturing of chemical products and materials, are playing a vital role in promoting economic development of the country and uplifting the living standards of citizens. But these industries are highly depended on coal consumption [7]. The major proportion of coal is consumed by these industries and they are the main drivers of the economic development in China [8]. On the other hand, the deteriorating environment has raised the public anxiety on health concerns. There are lots of international accords, such as the Paris agreement, designed for global climate change commitment, supplying further impetus to China’s environmental protection.

Under the pressure to achieve the sustainable development, China has faced with the challenge to alleviate high carbon emission but keep the sustainable economic growth at the same time. Facing with the severe air pollution problem, the report of the 19th National Congress of the Communist Party of China highlights that “we should adhere to the principles of quality first and efficiency first, take the supply-side structural reform as the main line, promote the quality reform, efficiency reform and power reform of economic development, and improve total factor productivity”. See http://cpc.people.com.cn/nl/2017/1115/c415067-29648367.html?ivk_sa=1024320u. Green technology innovation has thus become one of the key approaches to fight the battle of pollution prevention and promote the construction of ecological civilization.

Theoretically, the relation between environmental regulation and firms’ green innovation is two-sided. A lot of past research proposes that environmental regulation plays an important role in affecting corporate green innovation. For example, according to Porter’s Hypothesis, facing the pressure of stimulating innovation and technological process brought by the implementation of environmental regulation, firms further increase R&D investment and actively engage innovation, which is called a “leverage effect” [9]. However, according to Liu and Xiao (2022) [10], this effect may not be pronounced for all firms, especially for those with limited resources and severe regulation cost problems. Firms’ current resources (i.e. R&D investment, human capitals…) would be transferred from normal technological innovation to the solution to instantly decrease pollution emission to cope with new launched standard. Existing empirical studies have used pollution abatement control expenditures [11], prices of energy [12] and different types of policy instruments [13] to proxy for the stringency of environmental regulation, but most empirical studies on the effects of environmental regulation on green innovation are conducted at the macro or medium level, i.e. country level [14], provincial level [15] or industry level [16] with different conclusions.

With the rapid economic growth, China’s ecological imbalance and environmental pollution problems are also increasingly prominent. The problem of air pollution is particularly serious, which has become an important hidden danger threatening people’s lives and economic development. In response to the increasingly serious air pollution problem, China has continuously strengthened environmental policies. This has also triggered discussions on the economic effects. Referring to environmental policy we highlight in this paper, the *Ambient Air Quality Standard (2012)* is different from the existing mandatory environmental regulation policies such as voluntary emission reduction plan, energy regulation, government subsidies, environmental supervision and law enforcement [17]. It does not make enforcement measures, but enforce the pilot cities to disclosure the air quality to the public.

With the exaggerated information disclosure, the public in pilot cities improve the capability of obtaining environment related information, which reduces the information asymmetric between firms and the external stakeholders. The implementation of the *Ambient Air Quality Standard (2012)* makes it easier to obtain environmental information. The new standard requires that the urban air quality data be unified, real-time and comprehensively disclosed to the public. This can help to raise the government’s environmental concern. Further, it can also stimulate the public’s enthusiasm for environmental protection supervision, promote the adjustment of enterprise investment scale and structure, and form an environmental governance pattern of all the relevant stakeholders [18].

With the improvement of the transparency of air quality information, the real-time and uninterrupted disclosure of air quality status can provide a real and credible evaluation basis for local governments. This encourages governments to formulate green industrial policies and other environmental governance policies. And it can also urge financial institutions to adjust green financial policies and the optimization of other environmental governance policies.

To sum up, through the *Ambient Air Quality Standard (2012)*, the information of polluting firms become more transparency for superior governments, environmental protection departments, financial institutions, investors, the public and the media. The high information disclosure quality helps them to evaluate environmental risks of firms better.

This paper aims to test the impact of the implementation of environmental regulation on the green innovation of polluting firms, especially the public participation environmental regulation. And our study also engages to investigate the mechanism and the heterogeneity of the effect of environmental regulation on corporate green innovation. The *Ambient Air Quality Standard (2012)* provides a unique quasi-natural experimental setting for identifying the causal relationship between environmental regulation and green innovation, it includes three steps before spread nationwide. This new standard policy highlights the importance of air quality control. Thus, polluting firms are affected more by the new policy than other industries. This is the reason that we collect data of polluting firms as the sample. Moreover, there are three stages for the implication of the *Ambient Air Quality Standard (2012)*, which provides a natural experiment for trial cities selection and thus the implication of difference-in-difference approach. We use the sample of firms in the polluting industry listed in Shanghai and Shenzhen Stock Exchanges from 2007 to 2020 to investigate the impact of the *Ambient Air Quality Standard (2012)* on corporate green innovation.

This paper fills current research gaps in two ways. Firstly, compared with command control environmental regulations and market incentive environmental regulations, as a public participation environmental regulation, the *Ambient Air Quality Standard (2012)* plays the same role on including strengthening local government environmental protection intervention, promoting enterprise energy conservation and emission reduction, etc. This paper uses the PSM-DID method to eliminate the possible endogenous problems, empirically tested the impact of the *Ambient Air Quality Standard (2012)* on green innovation of polluting firms, and supplements the literature on the relationship between environmental regulations and corporate green innovation activities. Secondly, unlike the existing studies which focus on developed economies, this paper adopts a systematic measurement method to investigate the impact of environmental policy on corporate green innovation in the transformational economy, China. As the main body of promoting economic development, the study of the impact of air pollution control policies on firms’ green innovation has a far-reaching impact on economic development strategy.

The innovation of our study can be concluded in three aspects. First, a unique research setting is identified that enables testing of the causality between environmental regulation and corporate green innovation using quasi-experimental approaches. We select firms locate in pilot cities as the treatment group and other firms in the control group for using propensity score matching approach. This research design can mitigate omitted variables bias and other endogeneity issues. Second, compared to the macro and industry data, analyzing the firm-level economic consequences of environmental regulation can not only avoid aggregation biases but also provide an understanding of the micro-impact of environmental regulations. Finally, the study enriches the literature on environmental regulation and green innovation of firms, and the results have important implications for policy makers and managers to make optimal decisions.

The remainder of the paper is organized as follows. The second section introduces the institutional background of the *Ambient Air Quality Standard (2012)* and proposed the main hypotheses. The methodology is explained in the third section. Results and discussions of basic hypothesis, channels and heterogeneity analysis are in the fourth, fifth and sixth section, respectively. The last section concludes.

## Literature review

### Institutional background

The year 2012 can be regarded as a milestone in China’s environmental governance change. At the end of 2011, the US embassy in China monitored and released the air quality report with PM2.5 value in Beijing, which has aroused great concern of the society and brought greater pressure for the central government on environmental governance. On February 29, 2012, the Ministry of Environmental Protection and the General Administration of Quality Supervision of the People’s Republic of China jointly issued the *Ambient Air Quality Standard (2012)*.

Central government has begun to face up to and gradually pay attention to the problem of pollution, and strengthened environmental governance from environmental protection legislation and specific policy-making. But before 2012, local governments were still lack of sufficient incentives to govern the local environment. Since 2005, the central government has successively issued the *Decision on Implementing the Scientific Outlook on Development and Strengthening Environmental Protection*, the *Interim Provisions on Punishment for Violations of Laws and Disciplines in Environmental Protection*, and the *Opinions on Establishing an Assessment and Evaluation Mechanism for Party and Government Leading Groups and Leading Cadres to Promote Scientific Development*, involving and highlighting the great importance of environmental indicators to the economic development assessments and political promotion program. However, since the data of environmental quality was still not transparent enough, the change of assessment mechanism cannot effectively incentive local officials to carry out substantive environmental governance [19].

Before the launch of new standard, circumstances of air pollution and economic development were varies from city to city. The *Ambient Air Quality Standard (2012)* is the first nationally widely environment standard to highlight the transparent of pollution data, which requires the firm and government to expose a series of details such as equipment installation, data quality control and professional training. The external stakeholders are no longer just the passive information receiver, but also invited and involved in the information disclosure process. It extensively decreased information asymmetry among firms and the public.

There are three stages for the national-wide implementation of the *Ambient Air Quality Standard (2012)*. Firstly, in 2012, the key regions of Beijing-Tianjin-Hebei, Yangtze River Delta and Pearl River Delta, as well as provincial capitals implemented the new policy. In 2013, 113 key environmental protection cities and national environmental protection model cities became the implementation cities. Then the *Ambient Air Quality Standard (2012)* was implemented in all prefecture-level cities. Finally, from January 1, 2016, the *Ambient Air Quality Standard (2012)* came into force in the whole nation. Take the first stage as an example, on May 21, the Ministry of Environment Protection published the implementation plan for the first stage monitoring of the new air quality standard, which required that by the end of October 2012, all national network monitoring points in 74 pilot cities should complete equipment installation and carry out trial operation. Monitoring shall be executed according to the new air quality standards by the end of the year. And from January 1, 2013, the air quality data of the 74 trial cities should be available and exposed to the public, including superior government departments and the media.

### Hypothesis development

Based on the Institutional theory, social influence toward conformity shapes the behavior of organizations [20, 21]. Firms are more likely to carry out green innovation when facing stricter regulatory pressure from the government and normative pressure from non-governmental organizations [22]. When publishing new environmental regulation, enterprises are usually urged by the government to cut down pollutant emissions and energy consumption [23]. Through the *Ambient Air Quality Standard (2012)*, superior governments and environmental protection departments carried out environmental supervision and environmental accountability, financial institutions are able to evaluate the environmental risks of local enterprises, investors were able to evaluate the degree of cleaner production of local enterprises, the public can make environmental complaints to the related government department, and the media can more easily obtain environmental pollution information. Thus, with the decrease of information asymmetry, if the enterprise performs poorly in environmental protection, it is more likely to suffer the capital market punishment of stock price falling [24].

Before the *Ambient Air Quality Standard (2012)*, there are two main types of environmental regulations: controlled environmental regulations and incentive-based environmental regulations. In the implementation process of controlled environmental regulations, the mandatory enforcement of the regulations increase the production cost of enterprises and lead to the decline of their R&D investment in the short run [25]. While in the long term, with the pressure of the continuously increasing costs, firms will be more willing to engage in green innovation activities so as to reduce the emission at the source in order to survive and develop [26].

Incentive-based environmental regulations represented by emission trading will control emissions within a given range in the short term by purchasing quotas or reducing production, which leads to the transformation of resources from “production” to “pollution control” [27] and then crowd out the normal R&D expenditures of enterprises in the short term [16]. However, in the long run, enterprises are more likely to increase investment in green innovation to permanently alleviate the pollution emission and maintain their profits. Therefore, environmental regulation, whether controlling or encouraging will ultimately promote enterprises’ intention to innovate in terms of green innovation.

Some literature has proved that environmental regulation can really increase corporate green innovation from different perspective. Besides the *Ambient Air Quality Standard (2012)*, China’s carbon emissions trading pilot policy can induce green innovation [28] and Cleaner Production Audit (CPA) program can significantly increase green patent [29]. The establishment of environmental monitoring facilities, such as automatic air quality monitoring station [30], can improve firms’ green innovation. When facing environmental regulation, enterprises prefer to choose continuous investment in green innovation [31]. There are also some literature contributes to the basement of our research. They reveal the importance of synergy. Ouyang et al. (2022) [32] find that the strategic synergy between local and neighborhood environmental regulations can be an essential tool to improve green innovation efficiency and achieve sustainable development. And Tang and Li (2022) [33] prove that public participation can promote regional green innovation. Therefore, Environmental regulations are an effective way to encourage enterprises to carry our green innovation [34].

The main concerns of environmental regulations have negative impact on corporate green innovation is that the regulations increase the cost of firms to comply with the new rules. Previous literature points out that environmental policy affect more on polluting industry [26]. Zhang (2022) [35] also finds that environmental regulation has a negative effect on green innovation for highly-polluting firms but does not significantly impact those with low pollution.

In order to avoid additional costs and other environmental concerns, environmental regulations encourage polluting firms to shift to cleaner production technologies [14]. On the other hand, the *Ambient Air Quality Standard (2012)* does not enforce firms to decrease pollutant emissions or establish market rules for emissions quota trading. Thus, during the application of this standard policy, under the pressure of public and administrative supervision, polluting firms are more likely to involve in green innovation activities. Based on this, this paper puts forward the first hypothesis:

H1: The implementation of the *Ambient Air Quality Standard (2012)* has a positive impact on green innovation of polluting firms.

The Porter’s Hypothesis believes regulation can stimulate enterprises’ technological innovation, because the benefits brought by innovation are greater than the costs brought by the regulation [9]. Most previous studies that evaluate the effects of environmental regulations on firms’ green innovation mainly discuss from two perspectives: cost and technology [1, 15, 36]. From a cost perspective, the environmental regulations increase the exposure of firms to risks and potentially increasing cost of dealing with environmental pollution; and then increase the cost of products and services. However, for the implementation of the *Ambient Air Quality Standard (2012)*, firms face with the increase of information transparency, and undertake greater pressure of external supervision rather than enforcement charge for decreasing pollutant emissions.

From a technology perspective, firms have two choices under the new regulation or law: exit from polluting industries or actively adopt green technology to meet regulatory requirements. For firms in the polluting industry, it would be extremely expensive for them to give up their existing business due to the high associated costs. They need to give up their existing property, plant, and equipment (PPE) as well as their established customer base; furthermore, they will have to invest in new PPE and incur the costs of market development to enter a new industry. When firms choose to meet basic regulatory requirements, the costs of their goods and services increase because compliance costs influence their product market competitiveness. Therefore, it is more efficient for firms to actively explore green innovations, conduct related research and development (R&D) activities and apply green technology to offset the cost of legal compliance. The adoption of green technologies also reduces material consumption and emissions, and improves the input-output ratio. Thus, green innovation can lead to improved competitiveness and financial performance of firms [37].

Facing the pressure of stimulating innovation and technological progress brought by the implementation of the *Ambient Air Quality Standard (2012)*, enterprises may further increase R&D investment on the basis of existing innovation investment, recruit more tech experts to actively engage in green innovation activities, thus to produce a “leverage effect” on green innovation. Specifically, after the implementation of the *Ambient Air Quality Standard (2012)*, enterprises further increase R&D investment to enhance their green competitiveness [10]. Based on this, this paper puts forward the second hypothesis:

H2a: The implementation of the *Ambient Air Quality Standard (2012)* has a positive impact on R&D expenditures of polluting firms.
H2b: The implementation of the *Ambient Air Quality Standard (2012)* has a positive impact on the R&D personnel of polluting firms.

Environmental regulations can incorporate environmental factors into firms’ decision-making, so as to form constraints on firms’ pollution behavior. Therefore, environmental regulations can improve the enthusiasm of firms in participating in environmental protection activities and increase firms’ environmental protection investment [38]. Environmental protection investment provides financial support for firms’ green innovation, which can increase their green patent application [39]. Based on this, this paper puts forward the third hypothesis:

H3: The implementation of the *Ambient Air Quality Standard (2012)* has a positive impact on environmental protection investment of polluting firms.

## Data and methodology

### Sample and data

This study uses the sample of firms in the polluting industry. Polluting industry is defined by *Directory of Classified Management of Environmental Protection Inspection of Listed Firms*. listed on Shanghai and Shenzhen stock exchanges from the period of 2007 to 2020. The financial and accounting data of sample firms is captured from China’s Stock Market and Accounting Research (CSMAR) data vendor, and winsorized at 99 and 1 quantiles. This paper selects 2007 as the starting year because since 2007, firms have been required to implement new accounting system reform, thus to avoid problems of data inconsistency. The distributions of the sample by industry are reported in Table 1. As shown in the table, major industries involved in the research are *Chemical Raw Materials and Chemical Products Manufacturing* (24.1%), *Pharmaceutical Manufacturing* (23.4%) and *Rubber and Plastic Products* (7.7%).

**Table 1:** Sample distribution by industry.

Industry	No.	Pct.
Chemical raw materials and chemical products manufacturing	233	24.1%
Pharmaceutical manufacturing	226	23.4%
Rubber and plastic products	74	7.7%
Power, heat production and supply	73	7.6%
Nonferrous metal smelting and rolling processing	72	7.5%
Metal products	59	6.1%
Textile	35	3.6%
Ferrous metal smelting and rolling processing	35	3.6%
Papermaking and paper products	27	2.8%
Architectural decoration and other construction	24	2.5%
Chemical fiber manufacturing	22	2.3%
Coal mining and washing	22	2.3%
Nonferrous metal mining and dressing	22	2.3%
Petroleum processing, coking and nuclear fuel processing	18	1.9%
Others	24	2.5%
Total	966	100%

*Note*. This table presents the sample distribution of polluting firms by industry. The sample contains 966 polluting firms according to *Classified Management Directory of Environmental Protection Verification Industry of Listed Firms* from 2007–2020. Others include the following industries: oil and gas extraction; leather, fur, feather and related products and shoemaking; ferrous metal mining and dressing and non metallic ore mining and dressing.

## Variable selection

### Dependent variable

Our dependent variable, green innovation, is evaluated by the green patent applications of a firm in a given year. We collect all sample firms’ patent application details from 2007 to 2020 from IncoPat data vendor, then match the category of each patent with the detailed green innovation category of World Intellectual Property Organization (WIPO). Based on the research of Amore and Bennedsen (2016) [36], we eliminate design patent category since this category could not fully reflect firms’ innovation capability.

#### Independent variable

The independent variable of this paper is *Treat×Post*. This is the fixed independent variable of PSM-DID model. *Treat* is the dummy variable of the experimental group and *Post* is the dummy variable of the experimental period. In this paper, the experimental group refers to cities highlighted in the new standard and the experimental period is 2012 and after. The interaction term *Treat×Post* equals to 1 when the firm *i* is in the experimental group and enters the experimental period, otherwise equals to 0. *Treat* depicts the differences between the experimental group and the control group, *Post* depicts the differences between firm *i* before and after the experiment, and the interaction term *Treat×Post* measures the policy effect.

#### Control variables

This paper selects a set of control variables that may affect green innovation of enterprises, namely, firm size, firm age, firm leverage, firm performance [40, 41]. The natural logarithm of total assets is used to measure (in millions of RMB Yuan) firm size (*Size*). Firm age (*Age*) is the natural logarithm of listed years. This paper also controls for firm leverage (*Lev*) as the ratio of total debt to total assets, firm performance is sales growth rate (*Growth*). As mentioned at the second sector, the circumstances of economic development of different cities can affect the process of the implementation of the *Ambient Air Quality Standard (2012)*, therefore, we also introduce city-level control variables, namely, GDP and population, which are all in the logarithmic form.

### Empirical model

The time varying PSM-DID method can be used to effectively estimate the policy effect and alleviate the endogenous influence when the experimental group is not selected randomly. In reality, the policy is essentially a quasi-natural experiment (or non-randomized experiment), thus DID method can be used for policy effect evaluation. However, DID method inevitably has a self-selection bias. While using PSM-DID can avoid this kind of bias. The PSM method can match each treatment group sample to a specific control group sample when evaluating policy effect, making the quasi-natural experiment nearly random [42–44]. In order to make the cities of the experimental group and the control group as similar in all aspects as possible and eliminate the selection bias, we choose PSM-DID to accurately evaluate the effect of the impact of the *Ambient Air Quality Standards (2012)* on green innovation. This paper uses the policy of *Ambient Air Quality Standards (2012)* as a quasi-natural experiment to construct the PSM-DID estimation. This method first uses PSM method to match the research sample and then applies DID method to do the estimation. The first difference illustrates the policy shock, whether the firm is located in the city affected by the policy or not; and the second difference illustrates the time difference, whether it is before or after the launch of the new policy. Therefore, this study compares the difference of green innovation of polluting firms between pilot cities and non-pilot cities before and after the launch of the policy. The empirical model is as follows:

$$Green_{i,t}=\beta_{0}+\beta_{1}Treat\times Post+\sum Controls+\sum Industry\text{+}\sum City+\varepsilon_{i,t}$$ (1)

where *Green*_*i*,*t*_ refers to the green patent applications of polluting firms; *Treat* is a dummy variable equals to 1 if firm *i* is in the pilot cities and 0 otherwise; *Post* is also a dummy variable equals to 1 if year *t* is or after the city implement the new standard policy and 0 otherwise; the correlation coefficient *β*_*1*_ of *Treat× Post* is the policy effect of the *Ambient Air Quality Standard (2012)*; *Controls* refers to all control variables mentioned in the above section; *Industry* and *City* represent the industry fixed effect and city fixed effect, respectively; *ε*_*it*_ is the error term. The industrial distribution of the research sample reported are vary from Chemical Raw Materials and Chemical Products Manufacturing (24.1%) to Petroleum Processing, Coking and Nuclear Fuel Processing (1.9%), thus this study employs industry fixed effect to control the impact of industrial characteristic on green innovation. The economic development of different cities cannot only affect the policy implementation but can also affect the innovation activities, thus we also add city fixed effect into the regression.

In order to test the Porter Hypothesis, this study uses two-step method to prove the mechanism of R&D of the new standard policy affecting the green innovation of polluting firms, we also choose PSM-DID method to make the two-step method:

$$R\&D_{i,}{}_{t}=\alpha_{0}+\alpha_{1}Treat\times Post+\sum Controls+\sum Industry\text{+}\sum City+\varepsilon_{i,t}$$ (2)

$$Green_{i,}{}_{t}=\gamma_{0}+\gamma_{1}Treat\times Post+\gamma_{2}R\&D_{i,t}+\sum Controls+\sum Industry\text{+}\sum City+\varepsilon_{i,t}$$ (3)

where *R&D*_*i*,*t*_ represents the mechanism variable, R&D investment, which uses the percentage of R&D expenditures to total assets and the percentage of R&D personnel to total employees. When the *α*_*1*_ in the Eq (2) and *γ*_*2*_ in the Eq (3) are both significant different from zero, the mechanism is proved. The definitions of other variables are consistent with the above. The detailed definitions of variables are shown in the appendix.

This paper also uses two-step method to prove the mechanism of environmental protection investment of the new standard policy affecting the green innovation of polluting firms, we also choose PSM-DID method to make the two-step method:

$$EPInvest_{i,}{}_{t}=\alpha_{0}+\alpha_{1}Treat\times Post+\sum Controls+\sum Industry\text{+}\sum City+\varepsilon_{i,t}$$ (4)

$$Green_{i,}{}_{t}=\gamma_{0}+\gamma_{1}Treat\times Post+\gamma_{2}EPInvest_{i,t}+\sum Controls+\sum Industry\text{+}\sum City+\varepsilon_{i,t}$$ (5)

where *EPInvest*_*i*,*t*_ represents the mechanism variable, environmental protection investment, which uses the percentage of firms’ environmental protection investment to total sales to measure. Similarly, when the *α*_*1*_ in the Eq (4) and *γ*_*2*_ in the Eq (5) are both significant different from zero, the mechanism is proved. The definitions of other variables are consistent with the above. The detailed definitions of variables are also shown in S1 Table.

## Results and discussion

### Descriptive statistics

Table 2 presents the descriptive statistics of the variables. The mean value of *Green* is 0.278, and the minimum value, median value and maximum value are 0.000, 0.000 and 3.332 respectively. This result suggests exceed half polluting firms do not have green innovation output, which suggests the heterogeneity issue in the data sample. Table 3 shows the mean in our sub-samples based different firm characteristics, which suggest firms with big size and/or low financial constraints are more likely to conduct green innovation capability. The implementation of the *Ambient Air Quality Standard (2012)* includes three stages, so we uses three variables to proxy the pilot cities, namely, *Treat1*, *Treat*2 and *Treat3*. The mean values of these three variables are 0.551, 0.670 and 0.710, respectively. These values mean 55.1%, 67% and 71% of polluting firms locate in the pilot cities for the three stages. Next, we introduce the descriptive statistics of control variables. The mean values of firm characteristic variables such as *Size*, *Age*, *Lev*, *Growth* and *Labor* are 8.181, 2.882, 43.297, 11.924 and 7.846 respectively. The average values of city characteristic are 18.048 for *GDP* and 6.479 for *Population*.

**Table 2:** Descriptive statistics.

	Observations	Mean	S.D.	Min	Median	Max
***Green***	9398	0.278	0.654	0.000	0.000	3.332
***Treat1***	9398	0.551	0.497	0.000	1.000	1.000
***Treat2***	9398	0.670	0.470	0.000	1.000	1.000
***Treat3***	9398	0.710	0.454	0.000	1.000	1.000
***Size***	9398	8.181	1.904	0.000	8.186	12.364
***Age***	9398	2.882	0.309	2.079	2.890	3.584
***Lev***	9398	43.297	22.409	0.000	43.582	98.206
***Growth***	9398	11.924	28.669	-53.462	8.454	146.948
***Labor***	9398	7.846	1.218	4.779	7.783	10.928
***GDP***	9398	18.048	0.400	17.098	18.103	18.610
***Population***	9398	6.479	0.213	6.363	6.418	7.381

*Note*. *Green* is the natural logarithm of one plus the sum of green invention patent applications and green utility model patent applications. *Treat1* is a dummy variable that equals to one if a firm locates in a city which implemented *Ambient Air Quality Standard (2012)* in 2012, and zero if otherwise, *Treat2* is a dummy variable that equals to one if a firm locates in a city which implemented *Ambient Air Quality Standard (2012)* in 2013, and zero if otherwise, *Treat3* is a dummy variable that equals to one if a firm locates in a city which implemented *Ambient Air Quality Standard (2012)* in 2015, and zero if otherwise. *Size* is the natural logarithm of total assets (in millions of RMB Yuan), *Age* is the natural logarithm of listed years, *Lev* is the ratio of total debt to total assets, *Growth* is the sales growth rate, *Labor* is the natural logarithm of total employees, *GDP* is the natural logarithm of cities’ GDP (in ten thousands RMB Yuan), *Population* is the natural logarithm of cities’ population (in ten thousands persons).

**Table 3:** T-test for heterogeneity.

*Variables*	***Big size***		***Small size***		*T-statistics*
	Observations	Mean	Observations	Mean	
***Green***	4699	0.425	4699	0.143	0.282***
	***Low financial constraints***		***High financial constraints***		*T-statistics*
	Observations	Mean	Observations	Mean	
***Green***	4564	0.415	4834	0.161	0.254***

*Note*. Standard errors are in parenthesis.

*** *p*<0.01,

** *p*<0.05,

* *p*<0.1.

Big size and small size are distinguished by the median value of operating revenue. High financial constraints and low financial constraints are distinguished by the median value of WW index.

### Baseline regression results

Table 4 reports the regression results of the impact of the implementation of the new standard on the green innovation of polluting firms. Columns (1)–(3) are regression results without control variables and add firm-level control variables and city-level control variables gradually incorporate city fixed effect only, and columns (4)–(6) are regression results without control variables and add firm-level control variables and city-level control variables gradually incorporate industry fixed effect and city fixed effect. Both in the first three and the last three columns, we first investigate the policy effect on firms’ green innovation without control variables, and then we add firm-level control variables and city-level control variables step by step due to the firm-level control variables affect green innovation of enterprises more. Similarly, we control city fixed effect in the all six columns because the economic development of the pilot cities are very different compared to the industry differences. The coefficients of *Treat×Post* in columns (1)–(3) are positive and significant at 1% level. This finding suggests that the implementation of *Ambient Air Quality Standard (2012)* has effectively improved of green innovation of sample firms. After incorporating industry fixed effect, the coefficients of *Treat×Post* in columns (4)–(6) are still positively significant. This result strengthens the conclusion that the implementation of the new standard policy increases the green innovation of companies. These results support H1. To sum up, our study uses time varying PSM-DID to test the causality between environmental regulation and green innovation from micro level. And we have proved that the implementation of a public participate environmental regulation can increase firms’ green innovation. This also refers to the innovation of this paper.

**Table 4:** Basic regression results.

	(1)	(2)	(3)	(4)	(5)	(6)
	*Green*	*Green*	*Green*	*Green*	*Green*	*Green*
***Treat×Post***	0.135***	0.124***	0.062***	0.135***	0.094***	0.053**
	(0.019)	(0.023)	(0.022)	(0.018)	(0.022)	(0.022)
***Size***		0.046***	0.042***		0.059***	0.057***
		(0.010)	(0.010)		(0.011)	(0.011)
***Age***		-0.053	-0.127**		-0.025	-0.077
		(0.055)	(0.064)		(0.046)	(0.054)
***Lev***		0.000	0.001		-0.001*	-0.001
		(0.001)	(0.001)		(0.001)	(0.001)
***Growth***		-0.000	-0.000		-0.000	-0.000
		(0.000)	(0.000)		(0.000)	(0.000)
***Labor***		0.078***	0.079***		0.059***	0.059***
		(0.020)	(0.020)		(0.016)	(0.016)
***GDP***			0.144***			0.097***
			(0.033)			(0.032)
***Population***			-0.169***			-0.116***
			(0.040)			(0.038)
***Constant***	0.755***	-0.167	-1.441***	0.922***	-0.168	-1.020***
	(0.013)	(0.188)	(0.336)	(0.158)	(0.256)	(0.374)
***Industry FE***	No	No	No	Yes	Yes	Yes
***City FE***	Yes	Yes	Yes	Yes	Yes	Yes
***Observations***	9398	9398	9398	9398	9398	9398
***R*** ^***2***^	0.217	0.256	0.259	0.283	0.313	0.314
***Adjusted R*** ^***2***^	0.192	0.233	0.235	0.260	0.290	0.291

*Note*. Standard errors are in parenthesis.

*** *p*<0.01,

** *p*<0.05,

* *p*<0.1.

*Green* is the natural logarithm of one plus the sum of green invention patent applications and green utility model patent applications. *Treat×Post* is a dummy variable that equals to one if a firm locates in a pilot city and after implementing year, and zero if otherwise. *Size* is the natural logarithm of total assets (in millions of RMB Yuan), *Age* is the natural logarithm of listed years, *Lev* is the ratio of total debt to total assets, *Growth* is the sales growth rate, *Labor* is the natural logarithm of total employees, *GDP* is the natural logarithm of cities’ GDP (in ten thousands RMB Yuan), *Population* is the natural logarithm of cities’ population (in ten thousands persons).

### Robustness test

#### Placebo test

Traditional placebo test randomly disrupt the treated group and the control group, but it is not suitable for time varying PSM-DID. In this part, we first divide the research sample into several groups according to cities, and then select a year randomly from year variables in each city group as its policy time. And then re substituted into Eq (1) and save the coefficient of *Treat×Post*. We repeat the above random process for 1000 times. After random treatment, the effect of the *Ambient Air Quality Standard (2012)* on the improvement of green innovation of polluting firms in no longer significant and less than zero. The coefficient distribution of *Treat×Post* after random treatment is shown in S1 Fig.

#### Other models

This paper uses the natural logarithm of one plus the sum of green invention patent applications and green utility model patent applications as the proxy variable for green innovation in the main regression. For the robustness tests, we applies Tobit, Poisson model, Negative Binomial model to exam the impact of the *Ambient Air Quality Standard (2012)* on firms’ green innovation. Table 5 shows the results. The coefficient of *Treat×Post* is still positively significant, which supports the finding that the *Ambient Air Quality Standard (2012)* increases green innovation of enterprises.

**Table 5:** Robust regression results for Poisson and Negative Binomial model.

*Dep Var =*	(1)	(2)	(3)	(4)	(5)
	*Green*	*Green count*	*Green count*	*Green count*	*Green count*
***Treat×Post***	0.231**	0.613***	0.295***	0.535***	0.190**
	(0.095)	(0.192)	(0.111)	(0.033)	(0.081)
***Constant***	-9.107***	-9.835**	-13.401***	1.516**	-5.407***
	(1.774)	(4.796)	(3.278)	(0.699)	(1.569)
***Controls***	Yes	Yes	Yes	Yes	Yes
***Industry FE***	Yes	Yes	Yes	Yes	Yes
***City FE***	Yes	Yes	Yes	Yes	Yes
***Observations***	9398	9398	9398	9383	9383
***Pseudo R*** ^***2***^	0.195	0.601	0.161	0.602	0.191

*Note*. Standard errors are in parenthesis.

*** *p*<0.01,

** *p*<0.05,

* *p*<0.1.

Columns (1)-(5) are Tobit model, Poisson model, Negative Binomial model, Zero-inflated Poisson model and Zero-inflated Negative Binomial model, respectively. *Green* is the natural logarithm of one plus the sum of green invention patent applications and green utility model patent applications. *Green count* is the sum of green invention patent applications and green utility model patent applications. *Treat×Post* is a dummy variable that equals to one if a firm locates in a pilot city and after implementing year, and zero if otherwise. *Controls* are control variables which include *Size*, *Age*, *Lev*, *Growth*, *Labor*, *GDP* and *Population*. *Size* is the natural logarithm of total assets (in millions of RMB Yuan), *Age* is the natural logarithm of listed years, *Lev* is the ratio of total debt to total assets, *Growth* is the sales growth rate, *Labor* is the natural logarithm of total employees, *GDP* is the natural logarithm of cities’ GDP (in ten thousands RMB Yuan), *Population* is the natural logarithm of cities’ population (in ten thousands persons).

#### Change dependent variable to green innovation activities

The descriptive statistics of the variable *Green* reveal that more than half of the firms have no green patent applications. However, the maximum of Green is 3.332. To avoid some firms which have many green patent applications affect the research results, we introduce a dummy variable, *Green dummy*, to measure whether a firm has green patent applications or not. The dummy variable equals to one if a firm have at least one green patent application, and zero if otherwise. According to the characteristics of the new dependent variable, we use Logit and Probit model for the research. Table 6 shows results of the above two models. The coefficients of *Treat×Post* maintain positive and significant.

**Table 6:** Robust regression results for Logit and Probit model.

*Dep Var =*	(1)	(2)
	*Green dummy*	*Green dummy*
***Treat×Post***	0.250**	0.123*
	(0.116)	(0.064)
***Constant***	-9.826***	-5.779***
	(2.122)	(1.158)
***Controls***	Yes	Yes
***Industry FE***	Yes	Yes
***City FE***	Yes	Yes
***Observations***	8445	8445
***Pseudo R*** ^***2***^	0.213	0.212

*Note*. Standard errors are in parenthesis.

*** *p*<0.01,

** *p*<0.05,

* *p*<0.1.

Columns (1) and (2) are Logit model and Probit model, respectively. *Green dummy* is a dummy variable that equals to one if a firm have green patent applications, and zero if otherwise. *Treat×Post* is a dummy variable that equals to one if a firm locates in a pilot city and after implementing year, and zero if otherwise. *Controls* are control variables which include *Size*, *Age*, *Lev*, *Growth*, *Labor*, *GDP* and *Population*. *Size* is the natural logarithm of total assets (in millions of RMB Yuan), *Age* is the natural logarithm of listed years, *Lev* is the ratio of total debt to total assets, *Growth* is the sales growth rate, *Labor* is the natural logarithm of total employees, *GDP* is the natural logarithm of cities’ GDP (in ten thousands RMB Yuan), *Population* is the natural logarithm of cities’ population (in ten thousands persons).

### Mechanism analysis

The Porter Hypothesis holds that the return brought by innovation can make up for part of the cost of environmental regulations [9]. So facing the pressure of stimulating innovation and technological progress brought by environmental regulations, firms increase R&D investment on the basis of existing innovation investment, actively carry out green technology innovation and promote the promotion of green innovation on the basis of existing innovation activities, finally produce a “leverage effect” on green innovation. We use two variables to measure R&D investment of firms, the percentage of R&D expenditures to total assets and the percentage of R&D personnel to total employees, namely *RD expenditure* and *RD personnel*. The mechanism analysis results are reported in Table 7. The coefficient of *Treat×Post* in column (1) and the coefficient of *RD expenditure* in column (2) are both positive and significant the 1% level. This finding suggests that the implement of the *Ambient Air Quality Standard (2012)* increases the R&D expenditures, and the increasing R&D expenditures promote firms’ green innovation. And the Sobel test is positively significant which supports the R&D expenditures mechanism. The coefficient of *Treat×Post* in column (3) and the coefficient of *RD personnel* in column (4) are both positively significant. This result means that the implement of the new standard policy can increase the R&D personnel of companies, and the increasing R&D personnel encourages firms to do more green innovation activities. The Sobel test is also positive and significant, supporting the R&D personnel mechanism. These results support H2a and H2b. All in all, in this part we display the mechanism of how the environmental regulation promotes firms to engage green innovation. The results show these enterprises are inclined to increase R&D investment rather than saving costs when facing the *Ambient Air Quality Standard (2012)*. We further reveal this phenomenon by using the Porter Hypothesis. This is also the innovative contribution of our study.

**Table 7:** R&D mechanism regression results.

*Dep Var* =	(1)	(2)	(3)	(4)
	*RD expenditure*	*Green*	*RD personnel*	*Green*
***Treat×Post***	0.003***	0.042*	0.952***	0.048**
	(0.001)	(0.022)	(0.248)	(0.022)
***RD expenditure***		4.807***		
		(1.011)		
***RD personnel***				0.006***
				(0.002)
***Constant***	-0.126***	-0.315	-107.775***	-0.401
	(0.008)	(0.394)	(4.025)	(0.414)
***Controls***	Yes	Yes	Yes	Yes
***Industry FE***	Yes	Yes	Yes	Yes
***City FE***	Yes	Yes	Yes	Yes
***Observations***	9143	9143	9398	9398
***R*** ^***2***^	0.508	0.322	0.471	0.317
***Adjusted R*** ^***2***^	0.491	0.299	0.453	0.293
***Sobel***	0.017***		0.008***	
***Indirect effect***	0.014		0.006	
***Direct effect***	0.042		0.048	

*Note*. Standard errors are in parenthesis.

*** *p*<0.01,

** *p*<0.05,

* *p*<0.1.

*RD expenditure* is the percentage of R&D expenditures to total assets. *RD personnel* is the percentage of R&D personnel to total employees. *Green* is the natural logarithm of one plus the sum of green invention patent applications and green utility model patent applications. *Treat×Post* is a dummy variable that equals to one if a firm locates in a pilot city and after implementing year, and zero if otherwise. *Controls* are control variables which include *Size*, *Age*, *Lev*, *Growth*, *Labor*, *GDP* and *Population*. *Size* is the natural logarithm of total assets (in millions of RMB Yuan), *Age* is the natural logarithm of listed years, *Lev* is the ratio of total debt to total assets, *Growth* is the sales growth rate, *Labor* is the natural logarithm of total employees, *GDP* is the natural logarithm of cities’ GDP (in ten thousands RMB Yuan), *Population* is the natural logarithm of cities’ population (in ten thousands persons).

Wang and Wheeler (2005) [45] find that pollution levies are raised by a higher incidence of local pollution complaints. This finding proves that the public can promote the implementation of the *Ambient Air Quality Standard (2012)*. Due to the information disclosure theory, the firms’ best policy is to increase environmental protection investment in a short time [38]. And the raised environmental protection investment support green innovation activities of polluting firms [39]. We use the percentage of environmental protection investment to total sales to measure firms’ environmental protection investment, namely *EPInvest*. The mechanism analysis results are reported in Table 8. The coefficient of *Treat×Post* in column (1) and the coefficient of *EPInvest* in column (2) are both positive and significant the 1% level. This finding suggests that the implement of the *Ambient Air Quality Standard (2012)* increases the environmental protection investment, and the increasing environmental protection investment promote firms’ green innovation. And the Sobel test is positively significant which supports the environmental protection investment mechanism. These results support H3. In short, these findings of this section prove the information disclosure theory, referring to the increase of environmental protection investment when facing environmental regulation. This contribution also makes our study more in-depth.

**Table 8:** Environmental protection investment mechanism regression results.

*Dep Var =*	(1)	(2)
	*EPInvest*	*Green*
***Treat×Post***	0.001***	0.056***
	(0.000)	(0.021)
***EPInvest***		6.932***
		(2.508)
***Constant***	-0.003	-0.618
	(0.002)	(0.392)
***Controls***	Yes	Yes
***Industry FE***	Yes	Yes
***City FE***	Yes	Yes
***Observations***	8991	8991
***R*** ^***2***^	0.130	0.325
***Adjusted R*** ^***2***^	0.099	0.301
***Sobel***	0.008***	
***Indirect effect***	0.007	
***Direct effect***	0.056	

*Note*. Standard errors are in parenthesis.

*** *p*<0.01,

** *p*<0.05,

* *p*<0.1.

EPInvest is the percentage of environmental protection investment to sales. *Green* is the natural logarithm of one plus the sum of green invention patent applications and green utility model patent applications. *Treat×Post* is a dummy variable that equals to one if a firm locates in a pilot city and after implementing year, and zero if otherwise. *Controls* are control variables which include *Size*, *Age*, *Lev*, *Growth*, *Labor*, *GDP* and *Population*. *Size* is the natural logarithm of total assets (in millions of RMB Yuan), *Age* is the natural logarithm of listed years, *Lev* is the ratio of total debt to total assets, *Growth* is the sales growth rate, *Labor* is the natural logarithm of total employees, *GDP* is the natural logarithm of cities’ GDP (in ten thousands RMB Yuan), *Population* is the natural logarithm of cities’ population (in ten thousands persons).

### Heterogeneity analysis

#### Heterogeneity analysis for firm size

Large-scale firms are subject to higher costs of environmental regulation due to higher production. At the same time, their innovation costs are relatively lower due to the economies of scale. Thus, compared to small-scale firms, green innovation will be increased more in large-scale firms driven by internal benefit-cost tradeoffs [31]. Restricted by R&D personnel, R&D funds and other factors, it is more difficult for small-scale firms to carry out green innovation activities. Moreover, environmental regulations also lead to small firms more inclined to reduce R&D investment to enhance the end pollution control, so as to decrease green innovation activities. According to the median value of firms’ annual revenue, we separate total sample into two sub-samples: big size and small size for the heterogeneity analysis. The results are reported in Table 9. Columns (1) and (2) are the regression results for big firms while columns (3) and (4) are for small firms. The coefficients of *Treat×Post* in big firms are all positive and significant at 1% level. However, the coefficients of *Treat×Post* in small firms are not significant. This finding suggests that big size firms are more likely to participate in green innovation as response to the implementation of the new standard policy, while small firms might choose other approaches (i.e. decrease production) to decrease the polluted emission rather than involved in the innovation activities.

**Table 9:** Heterogeneity regression results by firm size.

*Sub sample* =	*Big size*		*Small size*	
	*Green*	*Green*	*Green*	*Green*
	(1)	(2)	(3)	(4)
***Treat×Post***	0.142***	0.122***	0.015	0.011
	(0.039)	(0.039)	(0.023)	(0.023)
***Constant***	-3.038***	-2.352***	0.792**	0.977***
	(0.655)	(0.691)	(0.316)	(0.321)
***Controls***	Yes	Yes	Yes	Yes
***Industry FE***	No	No	Yes	Yes
***City FE***	Yes	Yes	Yes	Yes
***Observations***	4699	4699	4699	4699
***R*** ^***2***^	0.293	0.377	0.219	0.240
***Adjusted R*** ^***2***^	0.256	0.342	0.175	0.194

*Note*. Standard errors are in parenthesis.

*** *p*<0.01,

** *p*<0.05,

* *p*<0.1.

Big size and small size are distinguished by the median value of operating revenue. *Green* is the natural logarithm of one plus the sum of green invention patent applications and green utility model patent applications. *Treat×Post* is a dummy variable that equals to one if a firm locates in a pilot city and after implementing year, and zero if otherwise. *Controls* are control variables which include *Size*, *Age*, *Lev*, *Growth*, *Labor*, *GDP* and *Population*. *Size* is the natural logarithm of total assets (in millions of RMB Yuan), *Age* is the natural logarithm of listed years, *Lev* is the ratio of total debt to total assets, *Growth* is the sales growth rate, *Labor* is the natural logarithm of total employees, *GDP* is the natural logarithm of cities’ GDP (in ten thousands RMB Yuan), *Population* is the natural logarithm of cities’ population (in ten thousands persons).

### Heterogeneity analysis for financial constraints

Capital investment is the first step of enterprise innovation and the key indicator of innovation success. The amount of capital investment could also reflect the importance, willingness and strength of enterprise to engage green innovation [34]. In the macro level research, the economic development level is usually considered as one of the factors that affect green innovation [46]. With the great risk and high investment of green innovation, it is difficult for companies with strict financing constraints to carry out green innovation activities. To test the impact of the *Ambient Air Quality Standard (2012)* on firms with different financial constrained levels, we divide the research sample into two sub-samples according to the median value of WW index. Table 10 reports the results. The coefficients of *Treat×Post* in columns (3) and (4) are both positive and significant at 1% level. While the coefficient of *Treat×Post* in columns (1) or (2) is only significant at 10% level or insignificant. This finding suggests that the impact of the new standard policy on green innovation is more prevalent in firms with low financial constraints.

**Table 10:** Heterogeneity regression results by financial constraints.

*Sub sample* =	*High financial constraints*		*Low financial constraints*	
	*Green*	*Green*	*Green*	*Green*
	(1)	(2)	(3)	(4)
***Treat×Post***	0.042*	0.034	0.130***	0.133***
	(0.023)	(0.023)	(0.042)	(0.041)
***Constant***	-0.072	0.064	-2.523***	-1.924***
	(0.361)	(0.367)	(0.658)	(0.690)
***Controls***	Yes	Yes	Yes	Yes
***Industry FE***	No	No	Yes	Yes
***City FE***	Yes	Yes	Yes	Yes
***Observations***	4834	4834	4564	4564
***R*** ^***2***^	0.223	0.237	0.292	0.383
***Adjusted R*** ^***2***^	0.177	0.189	0.250	0.344

*Note*. Standard errors are in parenthesis.

*** *p*<0.01,

** *p*<0.05,

* *p*<0.1.

High financial constraints and low financial constraints are distinguished by the median value of WW index. *Green* is the natural logarithm of one plus the sum of green invention patent applications and green utility model patent applications. *Treat×Post* is a dummy variable that equals to one if a firm locates in a pilot city and after implementing year, and zero if otherwise. *Controls* are control variables which include *Size*, *Age*, *Lev*, *Growth*, *Labor*, *GDP* and *Population*. *Size* is the natural logarithm of total assets (in millions of RMB Yuan), *Age* is the natural logarithm of listed years, *Lev* is the ratio of total debt to total assets, *Growth* is the sales growth rate, *Labor* is the natural logarithm of total employees, *GDP* is the natural logarithm of cities’ GDP (in ten thousands RMB Yuan), *Population* is the natural logarithm of cities’ population (in ten thousands persons).

## Conclusion

Fast economic growth in China has caused problems of environmental pollution and created great pressure on the ecological environment. Using *Ambient Air Quality Standard (2012)* as a quasi-natural experiment, this paper empirically tests the impact of the implementation of the public participation environment regulation on the green innovation of polluting firms. Firstly, this paper uses the time varying PSM-DID method to test the effect of the implementation of the new standard on the green innovation of polluting firms. The results show that the implementation of the new air standard significantly promotes the green innovation of polluting firms. A series of methodologies, such as placebo test and Tobit model, show that the results are robust. Our research supports Porter’s Hypothesis of leverage effect and proposes that the R&D investment and environmental protection investment are the potential influencing channel. In addition, when considering the heterogeneity at firm level, those with big size and low financial constraints are affected more by the implementation of the new standard policy.

Despite the high conceptual novelty of our study and its valuable contributions to the literature, it is not without limitations. First, although we eliminate design patent category from the measurement of green innovation, we do not make a distinction between invention patent category and utility model patent category. We regard these tow patent categories as the same in the empirical tests. However, the innovation capability of the above two categories is different, especially utility model patents sometimes are regarded as a strategic innovation for government subsidies or tax incentives [10]. Therefore, future studies are encouraged to make efforts to investigate whether the impact of environment regulation on different kinds of patent is the same. Secondly, our study is conducted in firms belong to polluting industries. We have not researched the impact of environmental regulation on firms in non-polluting industries and the effect of polluting industries become more environmental friendly on non-polluting industries. Thus, the developed model should be validated in other contexts in the future.

In addition to theoretical contributions, our study also offers important management insights for practitioners. For policy makers, it is suggested to promote the positive incentive effect of environmental regulation on green innovation. And policy makers should implement the innovation driven development strategy and improve the ability of green innovation from the aspects of content, technology and mode. Especially as the key link of green innovation, it is particularly important to improve the efficiency of green innovation from the technical level. At the same time, policy makers should also pay attention to promote the positive incentive effect of environmental regulation on alleviating firms’ financing constraints, and establish a platform for enterprise financing. Based on the additional empirical tests, we find firms with big size and low financial constraints respond more to the implementation of the new standard. Therefore, companies are advised to actively engage in green innovation activities according to the characteristics of their production and operation activities. We also recommend that companies see crisis as an opportunity rather than a threat. Creatively responding to sustainable development may result in unexpected benefits that have a long-lasting impact on performance.

## Supporting information

S1 Table Definition of variables and data source. (PDF)

S1 Fig Coefficient distribution of *Treat×Post* after random treatment. (TIF)

S1 Dataset (XLSX)
