# Supplementary material for: Environmental regulation and green innovation of polluting firms in China
Source: PLoS One. 2023 Mar 9;18(3):e0281303. doi: 10.1371/journal.pone.0281303 (PMC9997900; doi:10.1371/journal.pone.0281303)
Supplement: S1 Table — (PDF) [file pone.0281303.s001.pdf]

S1 Table. Definition of variables and data source.

Variables	Description	Source
Dependent variable		
Green	The natural logarithm of one plus the sum of green invention patent applications and green utility model patent applications.	Authors self-calculated based on data collected from IncoPat data vendor
Independent variables		
Treat×Post	A dummy variable that equals to one if a firm locates in a pilot city and after implementing year, and zero if otherwise.	Central Government Announcement
Treat1	A dummy variable that equals to one if a firm locates in a city which implemented *Ambient Air Quality Standard* in 2012, and zero if otherwise.	Central Government Announcement
Treat2	A dummy variable that equals to one if a firm locates in a city which implemented *Ambient Air Quality Standard* in 2013, and zero if otherwise.	Central Government Announcement
Treat3	A dummy variable that equals to one if a firm locates in a city which implemented *Ambient Air Quality Standard* in 2015, and zero if otherwise.	Central Government Announcement
Control variables		
Size	The natural logarithm of total assets (in millions of RMB Yuan).	CSMAR
Age	The natural logarithm of listed years.	CSMAR
Lev	The ratio of total debt to total assets.	CSMAR
Growth	Sales growth rate.	CSMAR
Labor	The natural logarithm of total employees.	CSMAR
GDP	The natural logarithm of cities' GDP (in ten thousands RMB Yuan).	Statistical Yearbook
Population	The natural logarithm of cities' population (in ten thousands persons).	Statistical Yearbook
