# Supplementary figures and images for: Environmental regulation and green innovation of polluting firms in China
Source: PLoS One. 2023 Mar 9;18(3):e0281303. doi: 10.1371/journal.pone.0281303 (PMC9997900; doi:10.1371/journal.pone.0281303)

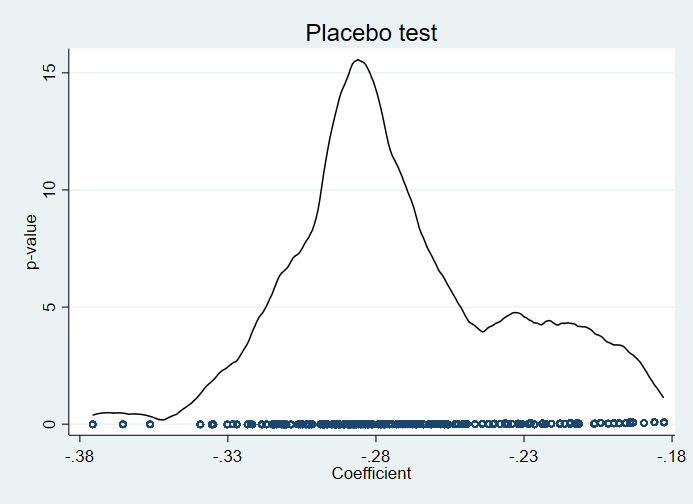

Supplement: S1 Fig — (TIF) [file pone.0281303.s002.tif]
